# Glial Endozepines Inhibit Feeding-Related Autonomic Functions by Acting at the Brainstem Level

**DOI:** 10.3389/fnins.2017.00308

**Published:** 2017-05-30

**Authors:** Florent Guillebaud, Clémence Girardet, Anne Abysique, Stéphanie Gaigé, Rym Barbouche, Jérémy Verneuil, André Jean, Jérôme Leprince, Marie-Christine Tonon, Michel Dallaporta, Bruno Lebrun, Jean-Denis Troadec

**Affiliations:** ^1^Laboratoire Physiologie et Physiopathologie du Système Nerveux Somato-Moteur et Neurovégétatif EA 4674, Faculté des Sciences et Techniques de St Jérôme, Université Aix-MarseilleMarseille, France; ^2^Institut National de la Santé et de la Recherche Médicale U1239, Laboratory of Neuronal and Neuroendocrine Communication and Differentiation, Institute for Research and Innovation in Biomedicine, University of Rouen NormadieMont-Saint-Aignan, France

**Keywords:** astrocytes, tanycytes, dorsal vagal complex, nucleus of the tractus solitarius, area postrema, food intake, swallowing, octadecaneuropeptide

## Abstract

Endozepines are endogenous ligands for the benzodiazepine receptors and also target a still unidentified GPCR. The endozepine octadecaneuropeptide (ODN), an endoproteolytic processing product of the diazepam-binding inhibitor (DBI) was recently shown to be involved in food intake control as an anorexigenic factor through ODN-GPCR signaling and mobilization of the melanocortinergic signaling pathway. Within the hypothalamus, the DBI gene is mainly expressed by non-neuronal cells such as ependymocytes, tanycytes, and protoplasmic astrocytes, at levels depending on the nutritional status. Administration of ODN C-terminal octapeptide (OP) in the arcuate nucleus strongly reduces food intake. Up to now, the relevance of extrahypothalamic targets for endozepine signaling-mediated anorexia has been largely ignored. We focused our study on the dorsal vagal complex located in the caudal brainstem. This structure is strongly involved in the homeostatic control of food intake and comprises structural similarities with the hypothalamus. In particular, a circumventricular organ, the area postrema (AP) and a tanycyte-like cells forming barrier between the AP and the adjacent nucleus tractus solitarius (NTS) are present. We show here that DBI is highly expressed by ependymocytes lining the fourth ventricle, tanycytes-like cells, as well as by proteoplasmic astrocytes located in the vicinity of AP/NTS interface. ODN staining observed at the electron microscopic level reveals that ODN-expressing tanycyte-like cells and protoplasmic astrocytes are sometimes found in close apposition to neuronal elements such as dendritic profiles or axon terminals. Intracerebroventricular injection of ODN or OP in the fourth ventricle triggers c-Fos activation in the dorsal vagal complex and strongly reduces food intake. We also show that, similarly to leptin, ODN inhibits the swallowing reflex when microinjected into the swallowing pattern generator located in the NTS. In conclusion, we hypothesized that ODN expressing cells located at the AP/NTS interface could release ODN and modify excitability of NTS neurocircuitries involved in food intake control.

## Introduction

In the early eighties, the search for an endogenous ligand for the benzodiazepine binding site of the GABA_A_ receptor led to the discovery of a precursor polypeptide named Diazepam-Binding Inhibitor (DBI; Costa et al., 1983; Guidotti et al., 1983; Corda et al., 1984). DBI and its proteolytic fragments octadecaneuropeptide (ODN; Ferrero et al., 1984) and triacontatetraneuropeptide (TTN; Slobodyansky et al., 1989) were collectively called endozepines (Tonon et al., 2013; Farzampour et al., 2015). DBI is identical to acyl-CoA-binding protein (ACBP, Knudsen, 1991), a cytosolic protein involved in fatty acid metabolism (Mogensen et al., 1987). It has been shown that astrocytes secrete DBI/ACBP through an unconventional pathway, in response to a variety of treatments (for review see Tonon et al., 2013; Farzampour et al., 2015).

Endozepines actually bind three molecular targets, the benzodiazepine binding site of the GABA_A_ receptor, also called Central-type Benzodiazepine Receptor (CBR; Guidotti et al., 1983; Tonon et al., 1989; Bormann, 1991; Louiset et al., 1993), the Peripheral-type Benzodiazepine Receptor (PBR), also called Translocator Protein (TSPO), a cholesterol transporter at the outer mitochondrial membrane (Berkovich et al., 1990; Papadopoulos et al., 1991; Gandolfo et al., 2001), and a still unidentified ODN G-protein-coupled receptor (ODN-GPCR; Patte et al., 1995; Gandolfo et al., 1997; Leprince et al., 2001).

The DBI gene is widely expressed in the central nervous system (Alho et al., 1989; Tonon et al., 1990; Costa and Guidotti, 1991; Malagon et al., 1993), with particularly high levels of expression in areas involved in food intake control such as the ventromedial and dorsomedial hypothalamus and the lateral hypothalamic area (Alho et al., 1985; Tonon et al., 1990; Malagon et al., 1993), suggesting a role of endozepines in food intake control. Accordingly, intracerebroventricular administration of ODN, or of its C-terminal octapeptide OP, in rodents exerts a potent anorexigenic effect (de Mateos-Verchere et al., 2001; do Rego et al., 2007; Lanfray et al., 2013). Moreover, intraparenchymal unilateral injection of OP in the arcuate nucleus of the hypothalamus also reduces food intake (Lanfray et al., 2013). The anorexigenic effect of endozepines does not depend on CBR or PBR signaling, but solely on ODN-GPCR signaling, since it is blunted by co-treatment with a selective ODN-GPCR antagonist (do Rego et al., 2007; Lanfray et al., 2013). Although, DBI mRNA and immunoreactivity has been found both in neurons and astrocytes (Alho et al., 1985, 1989; Tonon et al., 1990; Bouyakdan et al., 2015), DBI is mainly expressed in non-neuronal cells, such as ependymocytes, tanycytes, and proteoplasmic astrocytes (Tong et al., 1991; Lanfray et al., 2013; Bouyakdan et al., 2015). Consistent with a role of glial endozepines as anorexigenic factors, food deprivation reduces the mRNA expression of DBI in ependymocytes bordering the third and lateral ventricle as well as in median eminence tanycytes and arcuate protoplasmic astrocytes (Compère et al., 2010; Lanfray et al., 2013). The impact of endozepine signaling on neuronal populations involved in food intake control has been evaluated, with a focus on the hypothalamic leptin-sensitive neurons. Two populations of leptin-sensitive neurons located in the arcuate nucleus of the mediobasal hypothalamus play a major role in food intake control, one of which is inhibited by leptin and expresses the orexigenic neuropeptide Y (NPY), whereas the other one is activated by leptin and expresses the proopiomelanocortin (POMC), the endoproteolytic processing of which leads to the anorexigenic alpha-melanocyte stimulating hormone (α-MSH). It has been shown that central administration of ODN or OP reduces the expression of arcuate NPY mRNA, whereas it increases the expression of POMC mRNA (Compère et al., 2003, 2005). Moreover, pharmacological blockade of the melanocortin receptors type 3/4 (MC3/4R) abolishes the effects of OP on food intake, suggesting that the melanocortin signaling is a downstream effector of ODN-GPCR signaling in food intake control (Lanfray et al., 2013).

Up to now, the relevance of extrahypothalamic targets for endozepine signaling-mediated anorexia has been largely ignored. The dorsal vagal complex (DVC) located in the caudal brainstem is strongly involved in the homeostatic control of food intake. It comprises three interconnected structures: the area postrema (AP), a circumventricular organ, the nucleus tractus solitarius (NTS) and the dorsal motor nucleus of the vagus nerve (DMNX). Similarly to the arcuate nucleus of the hypothalamus, the NTS comprises a population of POMC-expressing neurons. Several lines of evidence showed that NTS POMC neurons mediate the acute anorectic effect of melanocortin signaling. NTS POMC neurons are activated by refeeding (Appleyard et al., 2005) and cholecystokinin (Fan et al., 2004). Moreover, their selective pharmacogenetic stimulation induces a rapid and strong anorectic effect (Zhan et al., 2013). By contrast, acute optogenetic (Aponte et al., 2011) or pharmacogenetic (Zhan et al., 2013) selective stimulation of arcuate POMC-neurons does not reduce food intake. Since endozepines mobilize melanocortin signaling, the DVC appears as a probable target site for endozepine-signaling-mediated anorexia. We previously studied the particularly dense glial coverage within the DVC. Interestingly, we described leptin-sensitive tanycyte-like cells form a barrier between the AP and the NTS and named these cells vagliocytes (Pecchi et al., 2007; Dallaporta et al., 2009, 2010). In the present study, we explored DBI expression profile within the DVC, as well as the effects of 4th ventricle administration of endozepines.

We show here that DBI is highly expressed in non-neuronal cell types within the dorsal vagal complex, with a strong labeling in ependymocytes and vagliocytes, and a more scattered labeling in protoplasmic astrocytes. Intracerebroventricular injection of ODN or its C-terminal octapeptide OP in the fourth ventricle reduces food intake in starved-refed rats treated in the light phase. This anorexigenic endozepine action goes with a cellular activation specifically located within the DVC. We also show that, similarly to other anorexigenic effectors acting at the brainstem level, microinjection of ODN into the swallowing pattern generator (SwCPG) located in the NTS inhibits the swallowing reflex elicited by stimulation of the solitary tract (ST). The swallowing reflex is a motor component of the alimentary canal involved in ingestive behavior. It allows the propulsion of the alimentary bolus from the oral cavity to the stomach.

## Animals

Experiments were performed on adult male Wistar rats (Janvier, France) of 250–350 g body weight. The animals were housed at controlled temperature on a 12:12 h light/dark cycle (lights on at 07.00 am) with food (SAFE, AO4) and water available *ad libitum*. Experiments carried out in this study were performed in strict accordance with European Economic Community guidelines (86/609/EEC) for the care and use of laboratory animals. The experimental procedures have been approved by our local Animal Care Ethics Committee (Comité Ethique de Provence N°13; license N° #2207-2015100819154639).

## Surgery and intracerebroventricular injection of endozepines

*Cannula implantation:* Animals were anesthetized by an intraperitoneal (ip) injection of ketamine (100 mg/kg; Imalgen 1000, Merial) and xylazine (6 mg/kg; Rompun, Bayer), and placed in a digital stereotaxic apparatus (Model 502600, WPI) coupled to the neurostar software (Neurostar GmbH). A 26-gauge stainless steel cannula was implanted into the fourth ventricle at the following coordinates: 12.7 mm posterior to bregma, 0.2 mm lateral to the midline, and 7.2 mm ventral to the skull surface. Verification of coordinates was performed by injecting 10 μl of China ink through the cannula. After rapid cryofreezing, 40 μm brain sections were realized and observed under an optic microscope (Nikon Eclipse E600W). The presence of the blue colorant in the walls on the 4th ventricle attested the right placement of the cannula. The right cannula placement was also checked at posteriori for each animal by histological observation of the cannula trace. The cannula was secured to the skull with dental cement and sealed with removable obturators. The animals were sutured, placed in individual cages and allowed to recover for 7 days. During this resting period, animals were injected with physiological saline every other day for habituation. One week post-surgery, rats were administered either 7 μl (2.2 μL/minute) of physiological saline, ODN or OP (2 μg/animal) solution 2 h after lights on. The correct cannula positioning was checked for each animal at the end of experiment by cresyl violet staining of brain sections. Subgroups of rats were anesthetized as previously described and perfused with paraformadehyde 4%, 90 min after injections for c-Fos procedures.

## Fast-refed experiments and food intake measurements

Rats were fasted for 20 h before being injected. Food was removed 6 h before lights off. Two hours after lights on, rat received either icv administration of ODN (*n* = 10), OP (2 μg/animal, *n* = 7) or vehicle (NaCl 0.9%, *n* = 12) as described above. The corresponding molar amounts for microinjection experiments of ODN (1912.13 g/mol) and OP (911.11 g/mol) are 1 nmol and 2.1 nmol, respectively. Forty-five minutes after treatment, a fresh supply of pre-weighed food was given and food intake was calculated as the difference between the pre-weighed and the remaining pellets measured with a precision balance (0.01 g; Denver Instrument from Bioblock) as previously described (Gaigé et al., 2014).

## Immunohistochemistry

Adult rats (*n* = 15) were deeply anesthetized with a mixture of ketamine (100 mg/kg ip; Merial, France) and xylazine (16 mg/kg ip; Bayer, France), transcardially perfused with 0.1 M sodium phosphate buffer (PBS; pH 7.4) and then, with freshly depolymerized 4% paraformaldehyde (PFA) solution in 0.1 M PBS. The brains were immediately removed, post-fixed 1 h in 4% PFA at room temperature and then cryoprotected for 24 to 48 h in 30% sucrose at 4°C. After freezing of the brains in cold-isopentane, coronal, horizontal and sagittal free-floating sections (30–40 μm thick) were cut on a cryostat (Leica CM3050, France) and rinsed in PBS. Sections were then treated with PBS containing 3% bovine serum albumin (BSA) to block non-specific binding sites and 0.3% Triton X-100. Sections were incubated overnight at 4°C with the respective primary antibody at 1/1000 (ODN: 403 2207 Tonon et al., 1990; GFAP: G3893, Sigma; vimentin: AB5733, Merck Millipore), washed in PBS and incubated for 2 h at room temperature with respective secondary antibody (1/200, Vector, CA, USA). Fluorescent images were acquired on a confocal microscope (Zeiss LSN 700) using the 488-nm band of an Ar-laser and the 543-nm band of a He/Ne-laser for excitation of FITC and TRITC, respectively. In double labeling experiments, images were sequentially acquired. All images were further processed in Adobe Photoshop 6.0; only contrast and brightness were adjusted and figures were not otherwise manipulated.

For c-Fos immunohistochemistry (*n* = 6), an anti-c-Fos rabbit antiserum (1/5000, Santa Cruz; SC-52) was used. Briefly, the free-floating sections were incubated 10 min in a solution containing 0.3% H_2_O_2_ in PBS 0.1 M for quenching of endogenous peroxidase activity. After 1 h in PBS containing 3% normal goat serum (NGS) and 0.3% Triton X-100, sections were incubated for 48 h at 4°C in PBS containing 3% NGS, 0.3% Triton X-100 and anti-c-Fos antibody. A biotinylated goat anti-rabbit IgG (1/400, Vector Labs) was used as secondary antibody. After incubation with the avidin-biotin complex (1/200, Vector Labs), horseradish peroxydase activity was visualized using a nickel-enhanced diaminobenzidine (DAB) as the chromogen. The reaction was closely monitored and terminated when optimum intensity was achieved (3–5 min) by washing the sections in distilled water. Three animals of each conditions and height sections per structure were analyzed. Non-specific labeling was assessed on alternate slices that were treated identically to the above but in which the primary antibody was omitted. c-Fos immunostaining photomicrographs were acquired using a 10-fold lens with a DXM 1200 Camera (Nikon) coupled to ACT-1 software. The microscope was set at a specific illumination level, as was the camera exposure time.

## Electron microscopy

Adult male Wistar rats (400–500 g, *n* = 6) were deeply anesthetized with a cocktail of ketamine (100 mg/kg ip; Merial, France)/xylazine (16 mg/kg ip; Bayer, France) and perfused by intraortic perfusion of 50 mL of 0.1 M PBS followed by 500 mL of 2% paraformaldehyde-1% glutaraldehyde in 0.1 M phosphate buffer. The brains were collected and immediately postfixed in the same solution for 3 h and then in 4% paraformaldehyde in 0.1 M phosphate buffer for 3 h. Coronal and sagittal serial sections of 50 μm thickness were made at the brainstem level using a Leica vibratome. The sections were treated with 1% sodium borohydride for 5 min. After thorough washes in PBS, they were successively incubated in 10% NGS for 30 min, in 10% NGS, 1% BSA and 0.1M lysine for 30 min, and then in rabbit anti-ODN antibody (#403; 1/800) overnight at 4°C. Following three washes, the sections were then transferred in a biotinylated goat anti-rabbit IgG (Vector Lab.) diluted 1/400 for 2 h at room temperature, washed thrice and incubated in the avidin biotin complex (Elite Vectastain kit, Vector Lab.) diluted 1/400 for 2 h at room temperature. Unless specified, the dilutions and washes between each of the above steps were made in PBS containing 1% NGS. The peroxidase activity was revealed with 0.003% of DAB and 0.01% H2O2. After that, the sections were post-fixed in 1% osmium tetroxide in 0.1 M phosphate buffer for 45 min, dehydrated in ethanol, and embedded in Epon. A portion of the DVC containing the interface between the AP and the NTS was then cut off under binocular. Ultrathin sections of ~70 nm in thickness were cut with an Ultracut ultramicrotome (Leica) and counterstained with uranyl acetate (5 min) and lead citrate (5 min). The ultrathin sections were then examined with a Philips CM 10 electron microscope (Center for Microscopy and Imaging of the Jean-Roche Institute) or JEOL JEM 2010F URP22 (Pluridisciplinary Center of Electron Microscopy and Microanalysis).

## Real time RT-PCR

DBI mRNA expression within the brainstem and the hypothalamus was examined as previously described (Gaigé et al., 2014). Briefly, total RNA was extracted from frozen brainstem and hypothalamus (*n* = 5) using Trizol. After RNA reverse transcription, gene expression analysis by real time PCR was performed using the ABI Thermocycler 7500 fast (Applied Biosystems). The equivalent of 6.25 ng initial RNA was subjected to PCR amplification in a 10 μl final volume using specific 2.4 μM primers and SYBR Green PCR master mix (Applied Biosystems). Product formation (DBI primers: Fw TGCTCCCGCGCTTTCA; Rev CTGAGTCTTGAGGCGCTTCAC) was detected at 60°C in the fluorescein isothiocyanate channel. The amplicon was then submitted to agarose gel electrophoresis to evaluate its size.

## Swallowing recording

Experiments were performed on adult male Wistar rats weighting 350 g (Charles River, I'Arbresle, France), anesthetized with 0.6 ml of a mixture of ketamine (100 mg/ml; Merial, France) and xylazine (15 mg/ml; Bayer France), injected intraperitoneally in a proportion of 90% and 10%, respectively. The anesthesia was then continued by perfusion of the same mixture diluted at 10%, through a catheter inserted in the femoral vein, at a rate of 0.01–0.05 mL/h. After occipitoparietal craniotomy and removal of the posterior part of the cerebellum, the floor of the fourth ventricle appeared to lie in a horizontal plane. The surface of the medulla was exposed in order to allow the stereotaxic introduction of the microelectrode in the intermediate NTS containing the SwCPG for ODN injection, and of the stimulatory bipolar electrode into the ST. The medulla was covered with warm liquid paraffin. Swallowing was triggered by central stimulation of the ST corresponding to the entering of the sensitive fibers which convey through the superior laryngeal nerve. Stimulation with a long train of pulses produced several swallows [or rhythmic swallowing recorded by electromyography (EMG)], at a rhythm depending on stimulation frequency. In the present study, repetitive long trains of pulses (5 s duration at 15 Hz frequency every 30 s) were used. The pulse voltage, duration and frequency varied according to the animal (2.6–4.8 V; 0.02–0.6 ms) in such a way as to trigger around 4–6 rhythmic swallows. To monitor swallowing, the EMG activity of sublingual muscles (mainly the geniohyoid) was recorded by means of bipolar copper wire electrodes inserted into the muscles, using a hypodermic needle. The respiratory activity was recorded by means of a mechanotransducer placed around the thorax, and the electrocardiogram (ECG) by subcutaneous electrodes placed on each side of the thorax. Moreover, the electrocardiogram and swallowing EMG signals were fed to loud speakers for auditory monitoring. Rectal temperature was monitored and maintained around 37°C with a heating pad. The EMG, ECG and respiration signals were recorded on a computer using an analog-to-digital interface (PowerLab 8SP data acquisition software for Windows, AD Instruments, USA). A stable control sequence involving three 30-s trains of stimulations was performed before ODN injection. The mean values obtained during this sequence were used as control values. Afterwards, stimulations and recordings were maintained until recovery. ODN injection in the SwCPG is a brief application. ODN was delivered in the SwCPG by pressure ejections through glass pipettes (70 μm O.D. at the tip) using an injection device (PMI-200, Dagan Corp., Minneapoli, MN USA). The pressure ejection was adjusted between 150 and 200 kPa for pulses of 3 s in duration, and the injected volume was 100 nl.

## Statistical analysis

Comparisons between groups in **Figure 6** were performed with repeated ANOVA with Tukey's HSD (Honestly Significant Difference) test used for *post-hoc* analysis. *P*-values < 0.05 were considered significant. Comparisons between groups in **Figure 7** were carried out with unpaired 2-tailed Student's *t*-test. *P*-values < 0.05 were considered significant. S.E.M values were derived from at three independent experiments. For swallowing experiment (**Figure 8**), statistical analyses were performed using one way analysis of variance (ANOVA) followed by Fisher's protected least-significant difference *post-hoc* test. Differences were considered significant when *P* < 0.05. Data were expressed as mean ± SEM. StatView for Windows 5.0.1; SAS Institute was used for statistical analysis.

## Results

### ODN immunoreactivity within the caudal brainstem is associated with glial cells

**Figure 1:**
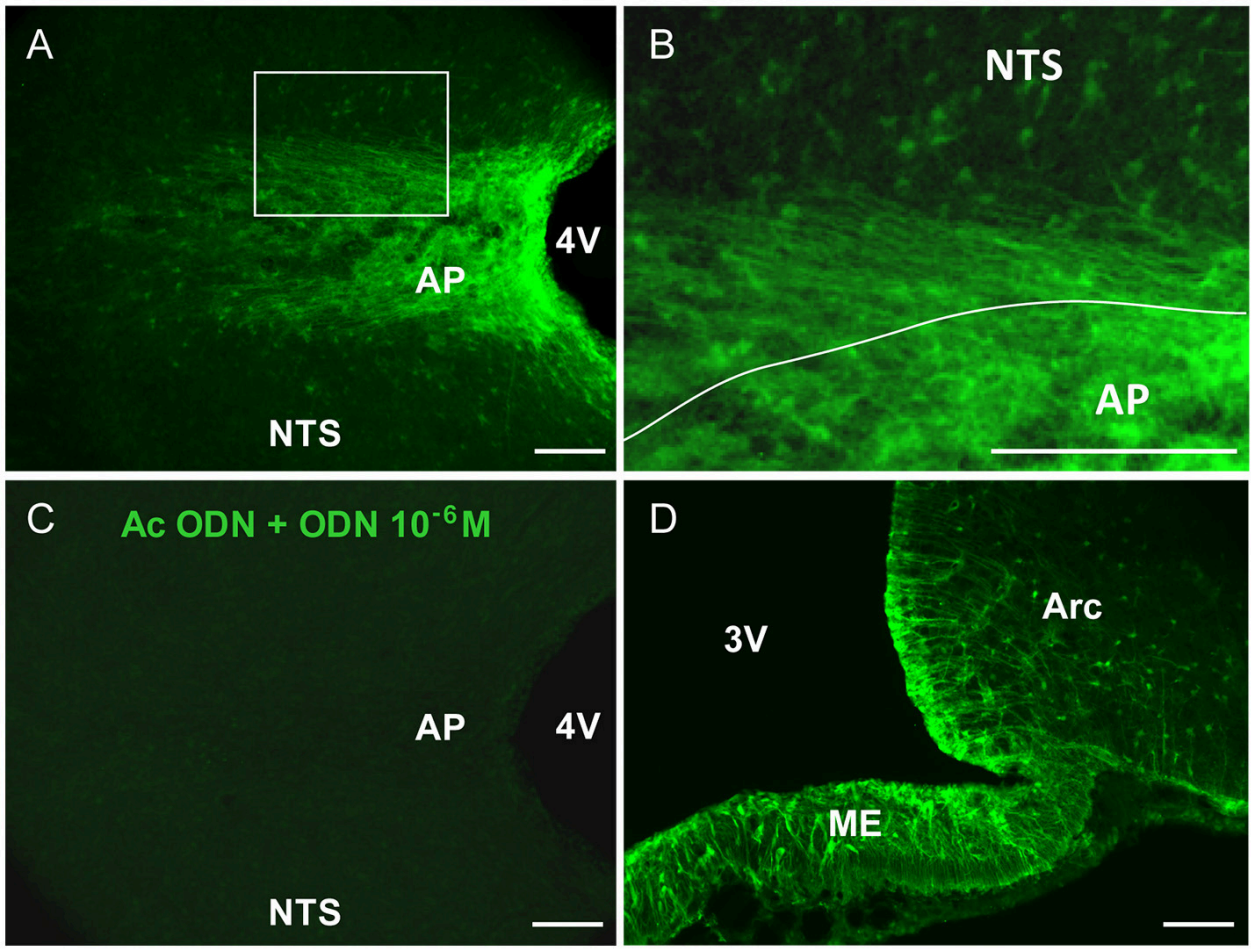
ODN immunohistochemistry in the brainstem. **(A)** Confocal fluorescent micrographs illustrating ODN immunohistochemistry performed on horizontal brainstem sections. ODN immunostaining showed the presence of positive cells in the area postrema (AP), at the border between AP and nucleus tractus solitarius (NTS) and within NTS surrounding the AP. Lateral dorsal vagal complex was devoid of labeling. **(B)** High magnification microphotograph originating from image in A (rectangle in A) and illustrating the shape of ODN immunoreactivity within the AP and surrounding structures. **(C)** Pre-incubation of ODN antibody with an excess of ODN peptide resulted in the absence of staining. **(D)** Confocal fluorescent micrograph illustrating ODN immunohistochemistry performed on coronal hypothalamic sections. As expected ODN immunoreactivity was found in the median eminence (ME), tanycytes lining the 3th ventricle (3V) and arcuate nucleus (Arc). 4V, 4th ventricle. Scale bars: 100 μm.

ODN immunohistochemistry within the rat brainstem, visualized on horizontal sections, revealed a striking pattern of immunoreactivity within the DVC, which distinguished it from the surrounding brainstem nuclei (Figure 1A). Within the DVC, a distinct subregional distribution of ODN immunoreactivity was observed. Indeed, the AP appeared heavily labeled throughout its rostro-caudal extent while a moderate ODN labeling was observed in the NTS (Figure 1A). Noticeably, the border between the AP and NTS i.e., the *funiculus separens* appeared also strongly stained (Figures 1A,B). At this level, a bundle of thin ODN-positive processes which radiated rostro-caudally into the NTS parenchyma was observed. Preincubation of the ODN antiserum with synthetic ODN (10^−6^ M) resulted in a complete loss of the immunoreaction (Figure 1C). Hypothalamic sections were used as positive control of ODN immunohistochemistry. As previously described (Lanfray et al., 2013), ODN staining was mainly observable in tanycytes lining the 3th ventricles and tanycytes located within the median eminence (Figure 1D).

**Figure 2:**
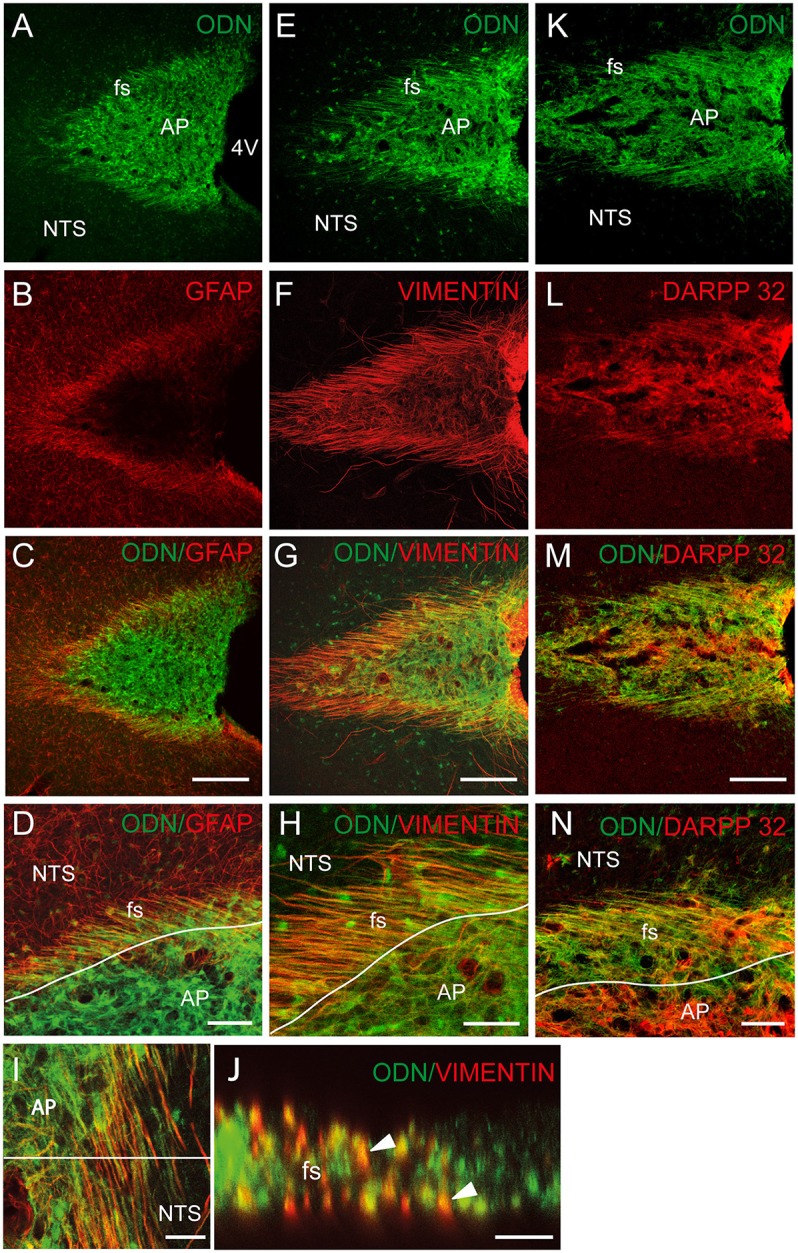
Tanycytes-likes cells of the brainstem are ODN positive. **(A–C)** ODN partly co-localized with GFAP immunostaining within the dorsal vagal complex (DVC). The presence of ODN/GFAP-positive processes was visualized at the nucleus tractus solitarius/area postrema (NTS/AP) border **(A–C)**. High magnifications illustrated the presence of ODN/GFAP double labeled radial processes **(D)**. **(E–H)** ODN partly co-localized with vimentin within the AP, at the AP/NTS interface i.e., *funiculus separens* (fs) and on the wall of the 4th ventricle (4V). **(I)** ODN/vimentin-positive processes originating from cells lining the 4th ventricle and radiating rostro-caudally were observed on horizontal sections of the DVC. A partial co-localization was also observed within the AP. **(J)** XZ orthogonal projection of images acquired on horizontal sections confirmed the presence of ODN/vimentin-positive processes surrounding the AP (arrowheads). **(K–M)** ODN/DARPP 32 co-localization within the AP and NTS/AP border. **(N)** High magnification illustrating ODN/DARPP 32 co-localization in long, radiating processes and cells located within the AP. Lines in D, H and N symbolize the AP boundary. Scale bars: 100 μm in **(A–C)**, **(E–G)**, and **(K–M)**; 40 μm in (**D,H,N**; 5 μm in **I** and **J**.

**Figure 3:**
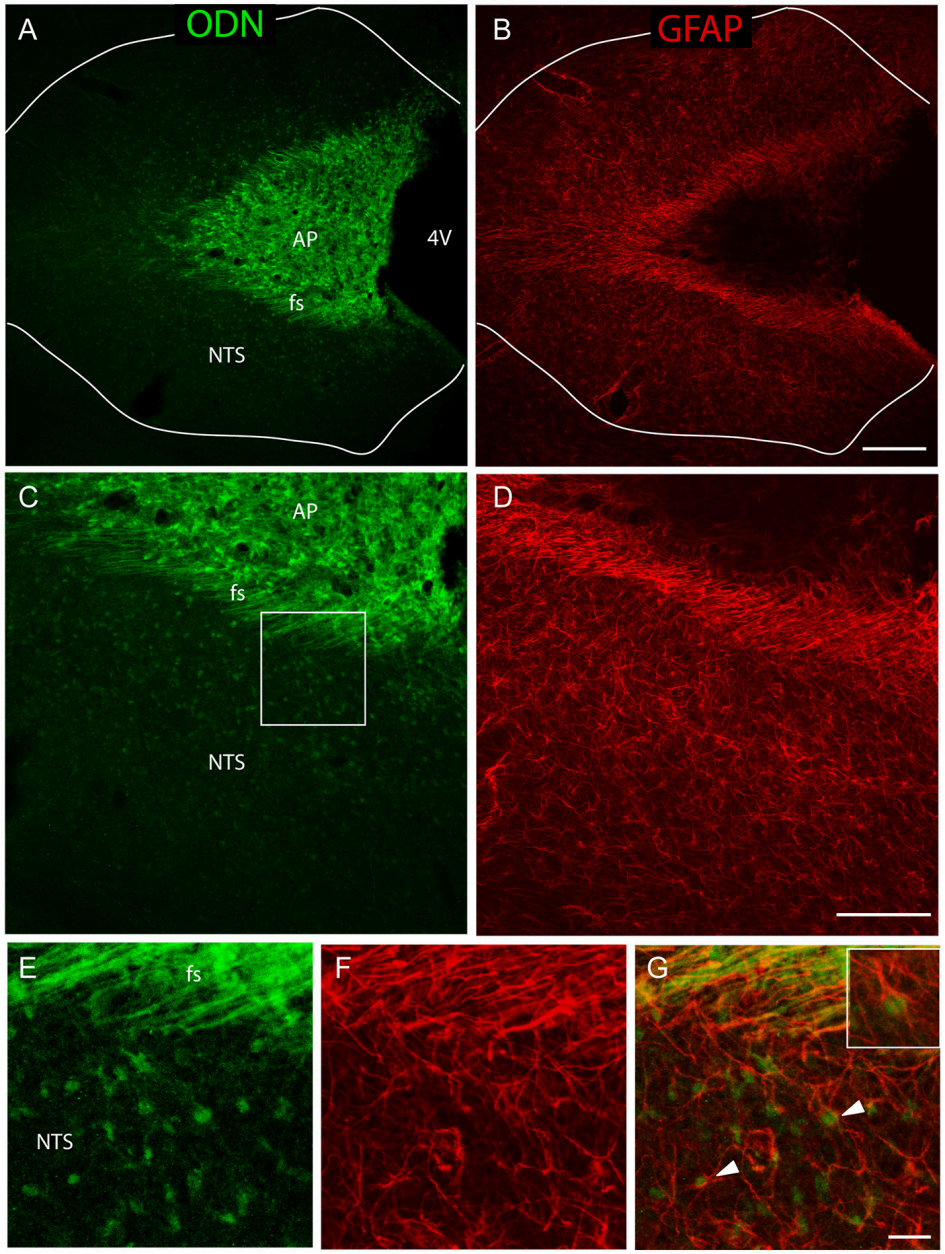
A sub-population of NTS protoplasmic astrocytes expressed ODN. **(A–D)** Low-magnifications images illustrated the presence of a rounded ODN labeling within the NTS. This labeling was intense in the vicinity of the *funiculus separens* and progressively diminished in the lateral parts of the NTS **(A–C)**. Similarly, the distribution of GFAP labeling was heterogeneous within the NTS, with a lesser concentrated staining in the lateral part than in the areas surrounding the *funiculus separens* **(B–D)**. Lines in A and B symbolize the NTS boundaries. **(E–G)** High-magnification images illustrated the presence of ODN/GFAP double labeled protoplasmic astrocytes located within the subpostremal NTS bordering the AP (arrowheads in **G**). Inset in **G**: Detail of an ODN positive astrocytes. Squares in **(C,D)** indicate the area from where images **(E–G)** originate. Scale bars: 250 μm in **(A–B)**; 100 μm in **(C–D)**, 10 μm in **(E–G)**.

To investigate the possible glial identity of brainstem ODN positive cells, we next performed ODN and GFAP double labeling. ODN-positive processes extending horizontally at the NTS/AP interface were also GFAP-positive (Figures 2A–D). Within the AP, ODN staining did not co-localize with GFAP since GFAP labeling was virtually absent from this structure. The presence of a sub-population of ODN/GFAP-positive cells exhibiting atypical morphology prompted us to perform vimentin immunolabeling since this intermediary filament protein is known to be expressed by tanycytes-like cells present at the brainstem level (Pecchi et al., 2007; Langlet et al., 2013). In the DVC, vimentin immunoreactivity was mainly observed in the NTS/AP border and on the edge of the 4th ventricle (Figures 2E–J). As expected, immunohistochemistry revealed the presence of ODN/vimentin immunoreactivity at the border between the AP and the NTS (Figures 2E–I). XZ orthogonal projection of images acquired on horizontal sections confirmed the co-localization of ODN and vimentin in thin processes surrounding the AP (Figure 2J). Within the AP, a part of ODN expressing cells remained vimentin negative, suggesting the existence multiple ODN-positive subpopulations in this structure. The dopamine- and cyclic adenosine-3′:5′-monophosphate (cAMP)-regulated phosphoprotein (DARPP-32) was found to be present in hypothalamic ependymal tanycytes lining the walls and floor of the third ventricle or located within the median eminence (Meister et al., 1988). At the AP level, DARPP-32 partly co-localized with ODN both within the AP and in the thin processes located in the *funiculus separens* (Figures 2K–N). In addition to ODN positive fibers located in the *funiculus separens* border area, ODN staining was observed within the NTS. This labeling exhibited a rounded shape and was observed in the vicinity of the *f. separens*. This ODN staining progressively diminished as one get away from the NTS/AP border (Figures 3A,C). Similarly, the GFAP labeling was heterogeneously distributed within the NTS, with a lesser concentrated staining in the lateral parts of the nucleus (Figures 3B,D). High-magnification images revealed an ODN/GFAP co-localization in cells exhibited typical features of differentiated protoplasmic astrocytes were observed (Figures 3E–G and inset in G).

### Electron microscopy of the NTS/AP border and ODN immunoreactivity

**Figure 4:**
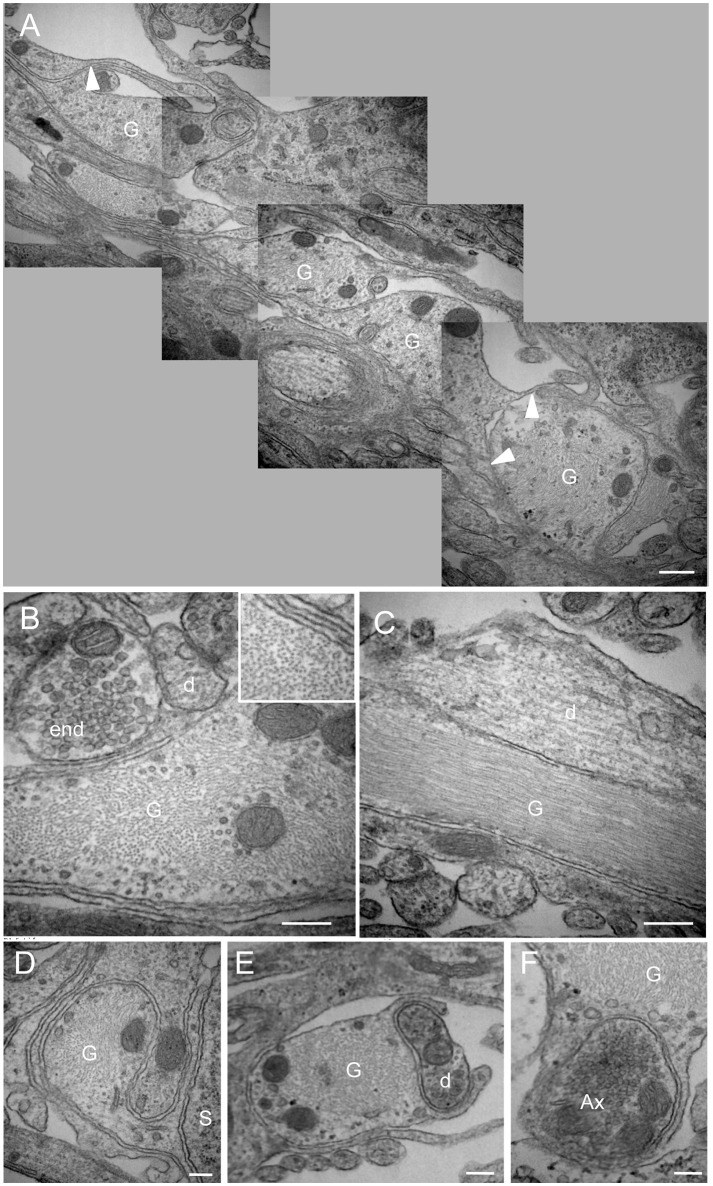
Morphological organization of the NTS/AP border. **(A)** Electron micrographs performed on sagital dorsal vagal complex sections showing the presence of rounded, fibrous glial processes at the border between the area postrema (AP) and nucleus tractus solitarius (NTS). Note that these fibrous processes create a continuous layer between the AP and the NTS. Small ramifications (arrowheads) of the rounded processes could be observed. **(B,C)** These glial processes could be identified by their numerous cytoskeleton filaments visible on sagital **(B)** and horizontal **(C)** sections. Inset in **(B)** Illustration of the high cytoskeleton filaments density in rounded glial processes. **(D–F)** Close juxtapositions between fibrous glial processes and neuronal elements i.e., neuronal soma **(D)**, dendritic profile **(E)** and axon terminal **(F)** are noticeable. Scale bars: 500 μm in A; 200 μm in **(B–F)**. D, dendritic profiles; Ax, axon terminals; S, soma; G: glial process.

**Figure 5:**
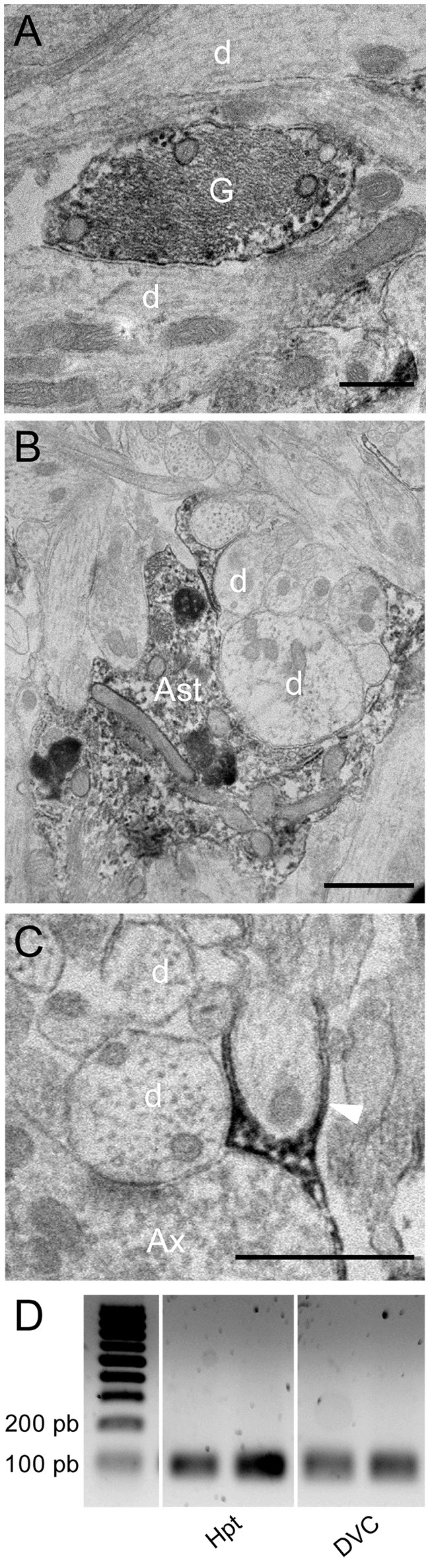
Immunoelectron microscopy of ODN. **(A,B)** Electron micrographs taken from the subpostremal nucleus tractus solitarius (NTS) illustrating the ODN immunostaining in protoplasmic astrocytes. Note that ODN positive processes are sometimes strongly associated with neuronal elements **(B)**. **(C)** Electron micrograph originating from NTS/area postrema border showed a fibrous glial process positive for ODN (arrowheads). **(D)** RT-PCR of DBI mRNA from hypothalamus (Hpt) and dorsal vagal complex (DVC) extracts. Scale bars: 500 nm in A; 1 μm in B and C. Ast, astrocytes; d, dendritic profiles; Ax, axon terminals; G, glial process.

We next performed electron microscopy at the NTS/AP border zone of coronal brainstem sections. Serial mapping with electron microscopy at low power magnification provided an overview of this area, and showed that it was organized with numerous glial processes (Figure 4A). Interestingly, these fibrous processes seemed to shape a continuous layer between the AP and the NTS. It seems likely that these processes observed in electronic microscopy match to the atypical and thin glial processes described above. This level of analysis revealed also that these glial processes originating from tanycyte-like cells located at the NTS/AP interface were rounded with very short ramifications (Figures 4A,D–F). These processes were clearly identified by numerous intracellular cytoskeleton filaments (Figure 4B and inset), exhibiting a compact organization with a similar orientation (Figure 4C). In some cases, short ramifications originating from fibrous processes were found in close apposition to neuronal elements such as somata, dendritic profiles, or axon terminals (Figures 4D–F). At the electron microscopic level, ODN immunoreactivity was also found exclusively in glial processes and cells (Figure 5). In addition to tanycyte-like processes located at the NTS/AP interface (Figure 5A), ODN staining was also found associated with protoplasmic astrocytes located in the subpostremal NTS (Figure 5B). In both cells types, ODN immunoreactivity was cytosolic. ODN-positive protoplasmic astrocytes were sometimes found in the close vicinity of synaptic profiles (Figure 5C).

Finally, the presence of DBI mRNA in rat DVC was investigated by RT-PCR analysis. A cDNA band of the expected size (95 bp) was detected in the reverse transcribed products from this structure. cDNA from hypothalamus, a structure known for its high ODN expression (Alho et al., 1985; Tonon et al., 1990; Malagon et al., 1993), were used here as a positive control (Figure 5D).

### Fourth ventricle endozepine administration reduced food intake

**Figure 6:**
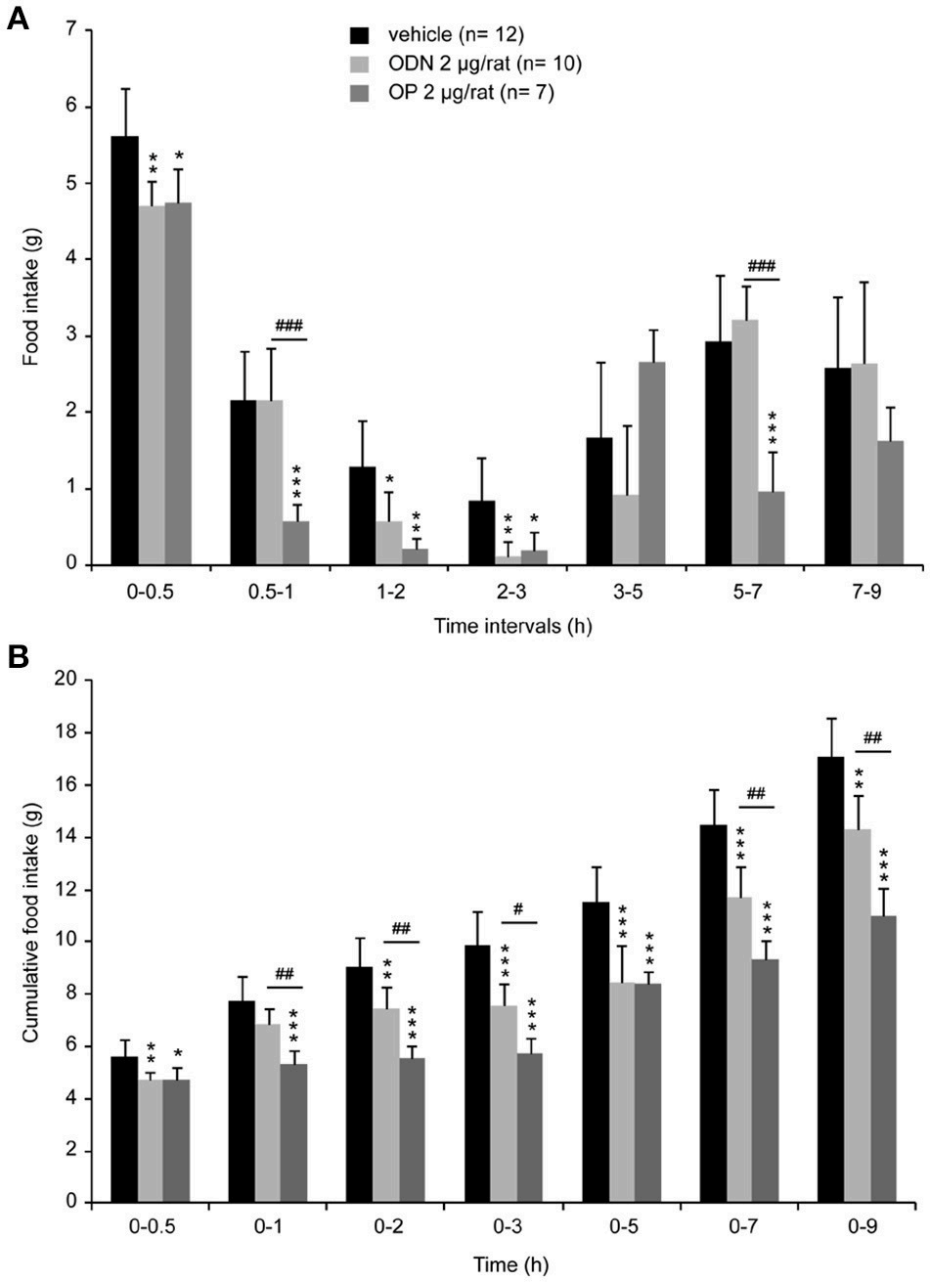
Acute 4th ventricle endozepine administration modifies food intake. Food intake measured at different time intervals after treatment **(A)** and cumulative food intake **(B)** monitored over 9 h after central injection of either saline (vehicle), ODN or OP (2 μg/rat). Animals were fasted 18 h before the 4th ventricle injection, and food was presented 45 min after treatment. ^*^, ^#^*P* < 0.05; ^**^, ^##^*P* < 0.01 and ^***^, ^###^*P* < 0.001 significantly different from vehicle-treated animals, respectively for ODN and OP-treated rats.

To determine whether endozepines could modify food intake by acting at the brainstem level, we performed 4th ventricle injection. The DVC which is involved in the initiation of meal as well as in the satiety reflex lines the 4th ventricle in the caudal brainstem. A single administration of ODN or OP (2 μg/animal) resulted in a decrease of food intake consumed during refeeding [Figure 6A, *F*_(2, 26)_ = 3.901, *P* = 0.0218]. This effect presented a short latency, since it was significant in the first hour post-treatment and feeding behavior remained profoundly affected during the first 3 h post-treatment. Cumulative food intake measured over a period of 9 h, revealed that OP affected food intake more deeply and durably than ODN [Figure 6B, *F*_(2, 26)_ = 18.006, *P* < 0.0001].

### Cellular activation induced by 4th ventricle OP administration

**Figure 7:**
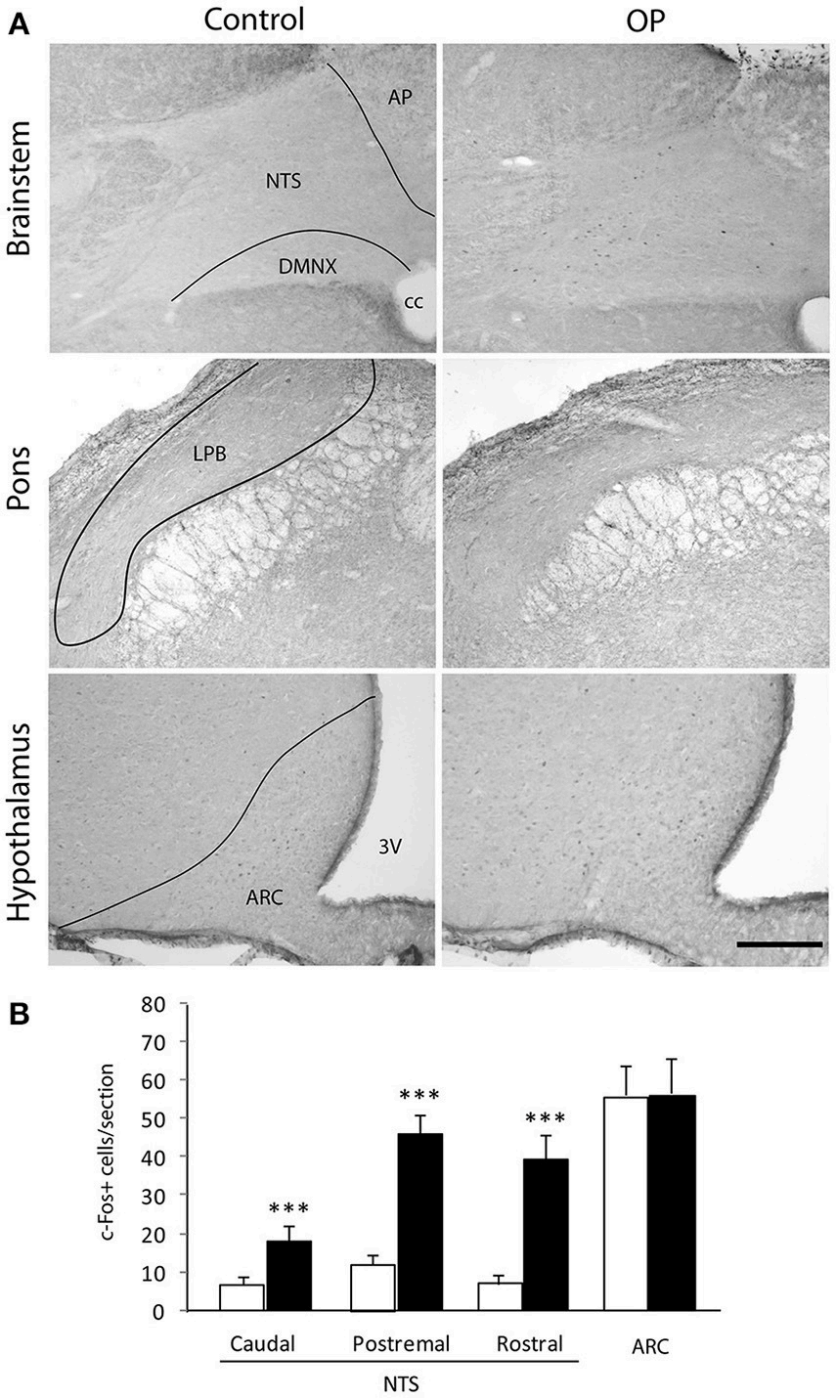
Effects of 4th ventricle OP administration on c-Fos immunoreactivity. **(A)** Representative coronal sections illustrating the c-Fos labeling observed within brainstem, pons and forebrain regions of mice treated with NaCl 0.9% (left panel) or anorexigenic OP dose (right panel, 2 μg/rat) and sacrificed 90 min post-treatment. **(B)** Quantification of the number of c-Fos immunoreactive nuclei within brainstem, pons and forebrain nuclei observed in mice treated either with vehicle (NaCl 0.9%, white bars) or OP (2 μg/rat, black bars). ^***^P < 0.001 significantly different from vehicle-treated rats. Scale bar: 150 μm. 3V, third ventricle, AP, area postrema; ARC, arcuate nucleus; cc, central canal; LPB, lateral parabrachial nucleus; NTS, nucleus tractus solitarius; DMNX, dorsal motor nucleus of the vagus.

We next sought to confirm that 4th ventricle OP injection resulted in cellular activation within the DVC and determine whether this cellular activation spread out of the brainstem. Central structures activated in response to OP (2 μg/animal) administration were identified using the immune detection of the c-Fos protein. Animals were sacrificed 90 min after injection of either NaCl 0.9%. or OP 2 μg/animal. A very low basal level of c-Fos positive nuclei was observed in the brainstem of NaCl-treated rats (Figure 7A). OP-treated rats exhibited a strong rise in the number of c-Fos positive nuclei within the NTS whatever the rostro-caudal level analyzed (Figure 7B). At the brainstem level, other DVC regions such as the AP and the dorsal motor nucleus of the vagus nerve (DMNX) were devoid of labeling (Figure 7A). At the time point analyzed here i.e., 90 min post-treatment, pontine and hypothalamic structures did not exhibited significant c-Fos expression increase in OP-treated animals as compared to control rats (Figures 7A,B).

### ODN inhibited swallowing reflex in anaesthetized rats

**Figure 8:**
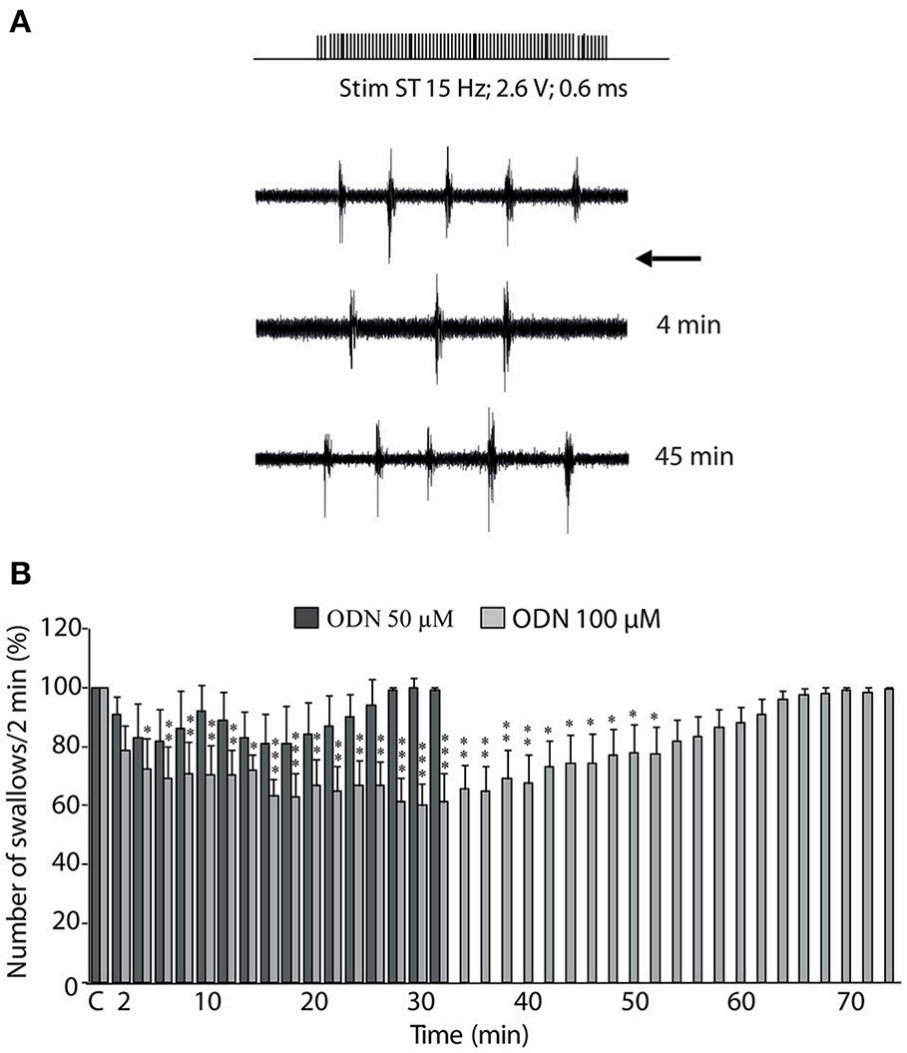
ODN inhibition of swallowing reflex. **(A)** Effects of ODN on sublingual muscle electromyographical activity induced by solitary tract (ST) stimulation. ODN (100 μM, arrow) injected within the swallowing pattern generator (SwCPG) induced a powerful inhibition of the number of swallows. Note that this inhibitory effect intervened rapidly after ODN application ~4 min. This effect was transient since the swallows recovered after 45 min. **(B)** Time course of the effects of ODN doses injected within the SwCPG at 50 μM (dark bars, 5 trials) and 100 μM (gray bars, 10 trials) on the number of swallows recorded over 2-min periods and until recovery. “C” represents the mean value of the control recorded before ODN microinjection (100%). Data are represented as means ± SEM normalized to the control value. ^*^P < 0.05, ^**^P < 0.01, ^***^P < 0.001 significantly different from control.

Given the pattern of OP-induced c-Fos expression observed within the DVC, we next tested the impact of a brief central ODN injection on the swallowing reflex. The present results showed ODN microinjections induced a dose-dependent decrease in the number of swallows recorded during ST stimulation (Figure 8A), with a slight non-significant effect at 50 μM (5 trials, 3 rats; Figure 8B), and a significant effect at 100 μM (10 trials, 5 rats; Figure 8B). The inhibitory effect observed upon 100 μM ODN microinjections appeared with a latency of 4.4 ± 1.4 min and persisted during 41.20 ± 4.96 min. This inhibitory effect of ODN on rhythmic swallowing pattern after its central injection within the SwCPG was not associated with variation of either cardiac frequency or respiratory activity (data not shown).

## Discussion

In the central nervous system, endozepines exhibit a wide distribution as reported by several groups (Alho et al., 1989; Tonon et al., 1990; Costa and Guidotti, 1991; Malagon et al., 1993). Indeed, using *in situ* hybridation or immunochemistry approaches, DBI or its major central processing product ODN was observed in many brain regions, such as olfactory bulb, cerebral, and cerebellar cortex, hippocampus, hypothalamus, inferior colliculus, and periaqueductal gray matter. Noticeably, very few data are available on brainstem DBI expression, with the exception of two *in situ* hybridization studies mentioning DBI expression within the AP (Alho et al., 1988; Tong et al., 1991). The present study was designed to perform a more comprehensive analysis of ODN expression at the brainstem level with a focus on the DVC. We observed a strong ODN expression in the AP, thus confirming previous observations (Alho et al., 1988; Tong et al., 1991). In addition, we reported a labeling in AP-surrounding regions i.e., the *funiculus separens* and the subpostremal/commissural NTS. From a functional point of view, the AP constitute a circumventricular organ of the brainstem necessary to relay the anorexic effects of circulating compounds such as amylin or leptin (Lutz et al., 2001; Liberini et al., 2016; Smith et al., 2016; Levin and Lutz, 2017). Furthermore, a subpopulation of AP neurons project heavily onto the immediately subjacent NTS i.e., subpostremal and commissural subnuclei (Shapiro and Miselis, 1985). The nervous peripheral input to the NTS through the vagus nerve exhibits a viscerotopographic organization (Loewy, 1990). The subpostremal and commissural NTS receive afferents from the gastrointestinal tract and integrate a wide variety of signals involved in the regulation of appetite and satiety (Shapiro and Miselis, 1985; Loewy, 1990). The brainstem ODN expression, we reported here, was thus associated with regions strongly involved in the integration of signals linked to the gastrointestinal tract and energy homeostasis.

We next sought to determine the cellular identity of ODN positive cells within the brainstem. Previous works using *in situ* hybridization have reported that in numerous brain areas, a specific labeling was associated with non-neuronal cells including ependymal and subependymal cells bordering the 3rd ventricle (Tong et al., 1991). By light microscope immunocytochemistry, ODN staining was also reported in glial and ependymal cells in the olfactory bulb, hypothalamus, hippocampus, periaqueductal gray, cerebral cortex, and the circumventricular organs (Alho et al., 1988; Tonon et al., 1990). These studies performed at the electron microscopic level confirmed the association of immunoreactive material with glial and ependymal cells (Alho et al., 1989). More recently, the identity of the endozepine-expressing cells within the hypothalamus was examined by immunohistochemistry. A strong ODN immunoreactivity was detected in DARPP 32/vimentin-positive thin processes, a labeling characteristic of tanycytes extending from the 3rd ventricle into the hypothalamic parenchyma (Lanfray et al., 2013). The results we obtained, at the brainstem level, showed that ODN and GFAP are co-expressed by protoplasmic astrocytes located in the subpostremal and commissural NTS subnuclei. Astrocytes from other parts of the NTS were devoid of labeling. In addition to ODN-positive astrocytes, ODN co-localized with GFAP in thin processes located within the *funiculus separens* known to originate from tanycytes-like cells i.e., vagliocytes previously described in this structure (Pecchi et al., 2007; Dallaporta et al., 2009). The cell bodies of these atypical glial cells are located at the border of the 4th ventricle or within the AP (Pecchi et al., 2007; Langlet et al., 2013). Co-staining of ODN with vimentin or DARPP-32 confirmed the ODN expression by DVC vagliocytes. The only partial overlapping between vimentin and DARPP-32 staining observed in the *funiculus separens* and the AP suggested that different sub-populations of vagliocytes co-exist within the DVC. Interestingly, these cells were reported to express leptin receptor (Dallaporta et al., 2009). Electron microscopy showed a cluster of ovoid fibers forming a continuous layer at the AP and NTS border. These processes were obviously identified by their numerous intracellular cytofilaments showing a similar orientation and a high density. These glial processes could exhibit small and short lateral ramifications. Interestingly, fibrous processes or their ramifications were sometimes found in close apposition to neuronal elements such as dendritic profiles or axon terminals. The localization and the shape of these processes are evocative of the transection of vagliocytes processes and previously visualized by immunohistochemistry. ODN staining observed at the electron microscopic level confirms its expression by vagliocytes and protoplasmic astrocytes. ODN expressing astrocytes were also occasionally found in the immediate vicinity of synapses. In total, these data show that, at the brainstem level, ODN is expressed by multiple glial cell populations mainly located at the AP/NTS interface. The localization of these ODN expressing cells at a neurohemal interface, together with their juxtaposition with neuronal components strongly lead us to put forward the hypothesis that ODN expressing cells could act as sensors of circulating compounds and could in turn release ODN and modify excitability of NTS neurocircuitries.

Intracerebroventricular administration of ODN or its C-terminal octapeptide fragment OP in the 3rd ventricle has been shown to exert a potent anorexigenic effect in rodents (de Mateos-Verchere et al., 2001; do Rego et al., 2007). Moreover, Lanfray et al. (2013) reported the inhibition of food intake after a unilateral injection of the ODN agonist OP into the arcuate nucleus, supporting the view that endozepines may control arcuate neurons involved in feeding behavior. The NTS is known as a primary integration site for satiety signals involved in the termination of a meal (Grill and Kaplan, 2002). Direct information about meal size arising from the gastrointestinal tract conveys through the vagus nerve to reach the NTS. Vagal mechanosensors located in the gastrointestinal tract sense the volume of ingested food and locally released satiety hormones, such as cholecystokinin (Schwartz and Moran, 1994; Berthoud et al., 2001). Despite, this major role of NTS in the termination of food intake, functional evidence for a role of endozepines in the brainstem regulation of feeding is missing. The brainstem ODN expression we reported here, led us to evaluate the impact of 4th ventricle endozepine injections on refeeding-induced satiety, a condition known to strongly mobilize the NTS (Timofeeva et al., 2005). We demonstrate here that 4th ventricle ODN or its C-terminal iso-active fragment OP (Leprince et al., 1998) injections strongly reduced food intake. OP was significantly more effective to reduce food intake than ODN at the same 2 μg dose. However, it should be noticed that the OP molar dose was twice that of ODN and partially explains this difference in cumulative food intake. This effect was observable rapidly after ODN or OP injection into the 4th ventricle, suggesting a local endozepine action restricted to the DVC region. This hypothesis was confirmed by c-Fos expression mapping since at a time point where OP reduced food intake, cellular activation was confined to the NTS, whereas more rostral structures and particularly endozepine-sensitive hypothalamic nuclei did not exhibit significant cellular activation. Reductions in food intake caused by the administration of exogenous compound must be cautiously interpreted because this could be secondary to aversion and induced sickness behavior. The involvement of the NTS and AP in the development of conditioned aversions and gastrointestinal malaise was clearly established. The activation of NTS and AP neurons was reported in response to a variety of aversive inputs and stressful stimuli (Yamamoto et al., 1992; Swank, 1999; Spencer et al., 2012. Interestingly, we did not observe any significant difference between elicited c-Fos immunoreactivity in saline- and OP-treated mice in the AP. Moreover, Grill and Norgren (1978) reported that decerebrates rats, in contrast to controls, neither rejected nor decreased ingestive reactions to a novel taste after that taste had been repeatedly paired with lithium chloride-induced illness supporting the idea that the forebrain may be important for taste aversion learning. Here, we did not observed c-Fos expression in forebrain structures after OP administration. Altogether, these data lessened the possible presence of an OP-induced sickness behavior. Nonetheless, this point should be experimentally addressed in the future.

Cerebroventricular OP injection resulted in a significant increase in c-Fos expression within the interstitial solitary tract nucleus subnucleus. This subnucleus is known to be involved in the control of respiratory, cardiac and swallowing autonomic functions. Swallowing, the first motor component of ingestive behavior, allows the propulsion of the alimentary bolus from the mouth to the stomach. Swallowing is triggered by sensory afferent fibers conveyed by the superior laryngeal nerve that project through the solitary tract to the DVC and premotoneurons located within the interstitial and intermediate NTS constitute the SwCPG (Jean, 2001). Anorexigenic and orexigenic factors have been shown to modulate the swallowing reflex. For instance, we have previously shown that different anorexigenic factors, such as leptin (Félix et al., 2006), the growth factor BDNF (Bariohay et al., 2008), and the mycotoxin deoxynivalenol (Abysique et al., 2015) inhibit the swallowing reflex. Concurrently, the orexigenic cannabinoids are reported to induce a facilitation of the swallowing reflex (Mostafeezur et al., 2012). This led us to conceive a possible modulation of the swallowing reflex by endozepines. The present study constitutes the first demonstration that endozepines could inhibit the swallowing reflex. We studied the effect of ODN microinjections within the SwCPG and we reported a transient but significant inhibition of rhythmic swallowing. Extracellular ODN microinjection used here may concern both passing axons and NTS neurons surrounding the injection site. ODN could indeed reach multiple neural components but the observed inhibitory effect appeared to be specific since (i) swallowing inhibition was never observed with NaCl alone and (ii) in our experimental protocol, other autonomic functions regulated at the brainstem level such as cardiac and respiratory functions remained unaffected. Hence, in the light of the present data, endozepines join the group of anorexigenic substances that both decrease food intake and inhibit swallowing.

In summary, the present work provides the first demonstration that endozepines are expressed by glial cells within the DVC. In this structure, ODN was found associated to protoplasmic astrocytes and tanycytes-like cells, located in regions known to integrate signals linked to the gastrointestinal tract and energy homeostasis. Moreover, the demonstration that endozepines affect food intake and swallowing reflex together with the close relationship of ODN expressing cells with neuronal elements strongly suggest that endogenous endozepines could act as local modulators of food intake behavior. The downstream ODN targets supporting the brainstem action of this peptide should be investigated in the future, with a particular attention to the melanocortin pathway (Lanfray et al., 2013).

## Author contributions

FG, CG, AA, SG, RB, JV, and MD performed, analyzed and interpreted data for the work. JL provided ODN and OP peptide, MT provided ODN antibody. MT, JL, MD, AJ, JT, and BL designed the work. JL, MT, BL, and JT wrote the paper. All authors revised the final version and approved it to be published.

### Conflict of interest statement

The authors declare that the research was conducted in the absence of any commercial or financial relationships that could be construed as a potential conflict of interest. The handling Editor declared a past co-authorship with one of the authors MCT, and the handling Editor states that the process met the standards of a fair and objective review.
